# Pyranose Dehydrogenase from *Agaricus campestris* and *Agaricus xanthoderma*: Characterization and Applications in Carbohydrate Conversions

**DOI:** 10.3390/biom3030535

**Published:** 2013-08-16

**Authors:** Petra Staudigl, Iris Krondorfer, Dietmar Haltrich, Clemens K. Peterbauer

**Affiliations:** Food Biotechnology Laboratory, BOKU–University of Natural Resources and Life Sciences Vienna, Muthgasse 11, Vienna 1190, Austria; E-Mails: petra.wuehrer@boku.ac.at (P.S.); iris.krondorfer@boku.ac.at (I.K.); dietmar.haltrich@boku.ac.at (D.H.)

**Keywords:** pyranose dehydrogenase, heterologous expression, agaricus, lactose conversion, lactobionic acid

## Abstract

Pyranose dehydrogenase (PDH) is a flavin-dependent sugar oxidoreductase that is limited to a rather small group of litter-degrading basidiomycetes. The enzyme is unable to utilize oxygen as an electron acceptor, using substituted benzoquinones and (organo) metal ions instead. PDH displays a broad substrate specificity and intriguing variations in regioselectivity, depending on substrate, enzyme source and reaction conditions. In contrast to the related enzyme pyranose 2-oxidase (POx), PDHs from several sources are capable of oxidizing α- or β-1→4-linked di- and oligosaccharides, including lactose. PDH from *A. xanthoderma* is able to perform C-1 and C-2 oxidation, producing, in addition to lactobionic acid, 2-dehydrolactose, an intermediate for the production of lactulose, whereas PDH from *A. campestris* oxidizes lactose nearly exclusively at the C-1 position. In this work, we present the isolation of PDH-encoding genes from *A. campestris* (Ac) and *A. xanthoderma* (Ax) and a comparison of other so far isolated PDH-sequences. Secretory overexpression of both enzymes in *Pichia pastoris* was successful when using their native signal sequences with yields of 371 U·L^−1^ for AxPDH and 35 U·L^−1^ for AcPDH. The pure enzymes were characterized biochemically and tested for applications in carbohydrate conversion reactions of industrial relevance.

## 1. Introduction

Pyranose dehydrogenase (PDH, EC 1.1.99.29) is a monomeric extracellular glycoprotein of around 75 kDa, carrying a covalently bound FAD cofactor (8α-N3-histidyl-FAD) [1]. It is a member of the glucose-methanol-choline (GMC) family together with other sugar oxidoreductases like the catalytically related enzymes glucose oxidase, cellobiose dehydrogenase and pyranose-2 oxidase. PDH was first described in 1997 when it was isolated from the edible basidiomycete fungus *Agaricus bisporus* [2]. Later, the enzyme from other members of the family of *Agaricaceae* like *Macrelepiota rhacodes* [3], *A. xanthoderma* [4] and *A. meleagris* [5,6] was investigated. Recently, the crystal structure of *A. meleagris* PDH (AmPDH) was resolved and revealed a two-domain structure consisting of the ADP-binding Rossman domain and a sugar-binding domain [1].

The biological function of PDH is still not fully clear. As the enzyme is limited to litter-decomposing fungi of the family *Agaricaceae* and is not able to utilize molecular oxygen as electron acceptor, the reduction of quinones and radicals formed during lignin degradation were proposed as its natural role [3]. Other possible functions like a participation in Fenton’s reaction or the defense against antimicrobial (quinone) substances produced by plants were reported [7].

As the production of the enzyme in basidiomycete fungi is quite laborious and time-consuming [2,4,5], approaches for heterologous expression in *Aspergillus nidulans* and *A. niger* [8], *E. coli* and *P. pastoris* [9] were tested. Attempts to solubly express PDH in *E. coli* did not succeed due to the formation of inactive inclusion bodies whereas an expression in *P. pastoris* yielded high levels of recombinant protein with properties equal to the wild-type [9]. Therefore, the methylotrophic yeast was the expression host of choice for the heterologous production of *A. campestris* and *A. xanthoderma* PDH in this study.

The oxidation products of PDH depend on the source of the enzyme, the substrate and the reaction conditions. The enzyme is able to oxidize free, non-phosphorylated sugars in pyranose form, heteroglycosides, disaccharides and glucooligosaccharides at the C-1, C-2, C-3 and also at C-1,2, C-2,3 and C-3,4 atom [2,3,10,11,12,13,14]. The oxidation products of D-glucose and D-galactose, 2-keto-D-glucose and 2-keto-D-galactose, represent industrially relevant intermediates for the production of the high-value sugars D-fructose and D-tagatose [15,16]. An easily available disaccharide lactose, can be oxidized by PDH to the corresponding C-1, C-2 or C-2,3 product. Depending on the source of the enzyme, 2-keto-lactose and lactobionic acid, the hydrolysis product of lactobionolactone, are formed in different ratios. Volc and coworkers screened various *Agaricus sp.* for their PDH oxidation products and observed that *A. campestris* PDH almost exclusively oxidizes lactose at the C-1 position, yielding lactobionic acid, whereas 2-keto-lactose was the main product of *A. xanthoderma* PDH [12]. Lactobionic acid has numerous applications in the pharmaceutical-, cosmetic- and food-industry, such as in organ preservation solutions and macrolide antibiotics, skin care cosmetics or as an acidulant or flavor enhancer in food [17]. The C-2 oxidation product 2-keto-lactose can be used for the production of lactulose, a prebiotic carbohydrate administered against obstipation and hepatic encephalopathy which has beneficial effects on the gastrointestinal microbiota [18]. Here, we describe for the first time the heterologous expression of PDH genes from *Agaricus campestris* and *Agaricus xanthoderma* in the methylotrophic yeast *Pichia pastoris* and present a detailed characterization of both enzymes. Furthermore, we performed comprehensive studies on the conversion of lactose and present a novel alternative for the production of lactobionic acid and 2-dehydrolactose, a key intermediate for the isomerization to lactulose.

## 2. Results and Discussion

### 2.1. Expression of Acpdh and Axpdh in P. pastoris

To obtain the PDH-encoding gene from *A. campestris,* different oligonucleotide primers were designed based on conserved regions from already known sequences (accession numbers are given in paragraph 3.8.). PCRs were performed using different forward primers, the float primer and first-strand-cDNA as template. Resulting fragments were sequenced and used to design sequence specific primers for identification of the 5‘-flanking region by primer walking using the DNA Walking SpeedUp Premix Kit. The nucleotide sequence of the AcPDH cDNA contains an ORF of 1,788 bp encoding a polypeptide of 595 amino acids. Two primers based on the cDNA sequence and containing restriction sites for ligation into the pPICZb vector were designed, and used to re-amplify the cDNA and construct the expression vector under control of the methanol-inducible AOX promoter. 

The previously unknown signal sequence and the 5‘-flanking region of AxPDH were analogously identified using the DNA Walking SpeedUp Premix Kit and three specific reverse primers (AxTSP1-3). The purified fragments were sequenced, and based on these results, the full-length cDNA could be amplified. The AxPDH encoding cDNA contains an ORF of 1,803 bp encoding a polypeptide of 600 amino acids. The cDNA fragment was re-amplified with two primers containing restrictions sites for ligation into the pPICZb vector. The plasmids were transformed into *E. coli* NEB5α for proliferation. Isolated plasmids were linearized with *SacI* and transformed into the expression host *P. pastoris* and cultivated in 96-well deep well plates and screened for PDH activity. To confirm the results from the first round of screening, a rescreening experiment with multiple parallel determinations was performed. The clones with the highest activity were selected for further studies.

### 2.2. Multiple Sequence Alignment

To compare the amino acid sequences of the PDHs isolated and characterized so far in our group (*A. meleagris* PDH1 [5,6,9], *A. bisporus* (our unpublished information) *A. campestris* (this work) and *A. xanthoderma* PDH [4]), a multiple sequence alignment was constructed using the MUSCLE algorithm (Figure S1). The PDHs from different sources show a sequence identity between 74% and 78%. From the crystal structure of AmPDH1 [1], His 512 and His 556 were identified as the catalytic pair of major importance for sugar substrate oxidation. These two amino acids and His 103, where the FAD cofactor is covalently bound, are highly conserved among PDHs (Figure S1, highlighted in red). Docking experiments with several electron donors in different oxidation poses revealed that the principal sugar interaction partners are the two catalytic histidines but also Gln 392 and Tyr 510 (Figure S1, highlighted in green). The fact that these amino acids are conserved in all four PDHs supports these findings. 

All PDHs are glycoproteins with different degrees of glycosylation. Putative N- and O-glycosylation sites predicted by NetNGlyc 1.0 server and NetOGlyc 3.1 server [19] are highlighted in red and green, respectively.

### 2.3. Heterologous Protein Production

**Figure 1:**
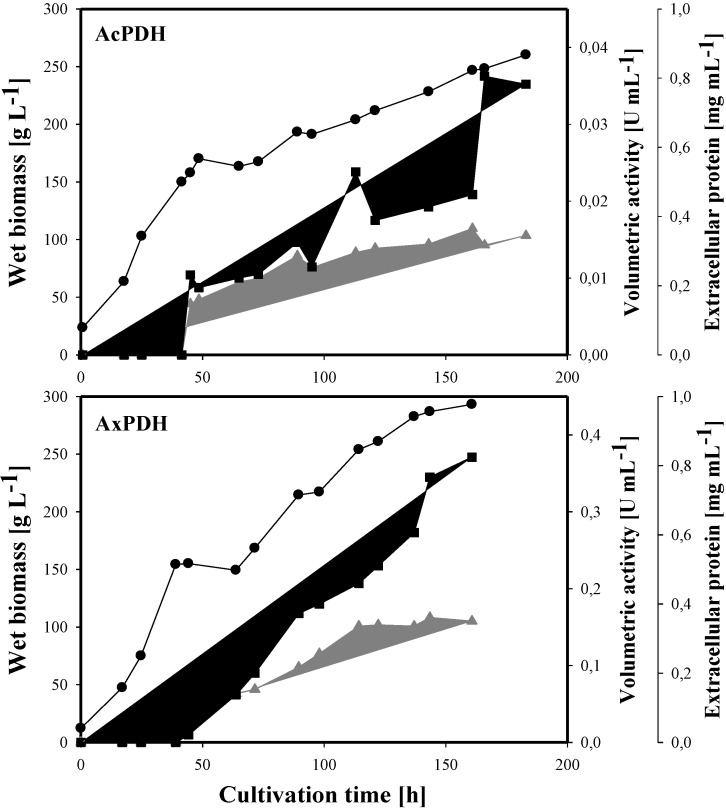
Large scale production of pyranose dehydrogenases (PDHs) in *P. pastoris*. Black circles, wet biomass; grey triangles, extracellular protein concentration; black squares, volumetric activity.

The cultivation of *P. pastoris* cells expressing the *acpdh*- and *axpdh*-encoding gene was carried out in a 7-L stirred and aerated bioreactor and lasted 187 and 161 h, respectively (Figure 1). The initial glycerol batch phase produced 113 g·L^−1^ and 75 g·L^−1^ wet biomass in 27 h and 25 h (its end was indicated by an increase in dissolved oxygen concentration). The following feed with 50% glycerol was maintained for 17 h and 19 h and resulted in a final biomass of 156 g·L^−1^ and 155 g·L^−1^, respectively. A methanol feed was initiated for induction, and at the end of this phase, the biomass reached a level of 260 g·L^−1^ and 293 g·L^−1^. The activity of the extracellular enzyme fraction finally reached 35 U·L^−1^ for AcPDH and 371 U·L^−1^ for AxPDH, while the level of extracellular protein increased to 345 mg·L^−1^ and 350 mg·L^−1^.

### 2.4. Purification of Recombinant PDHs

**Figure 2:**
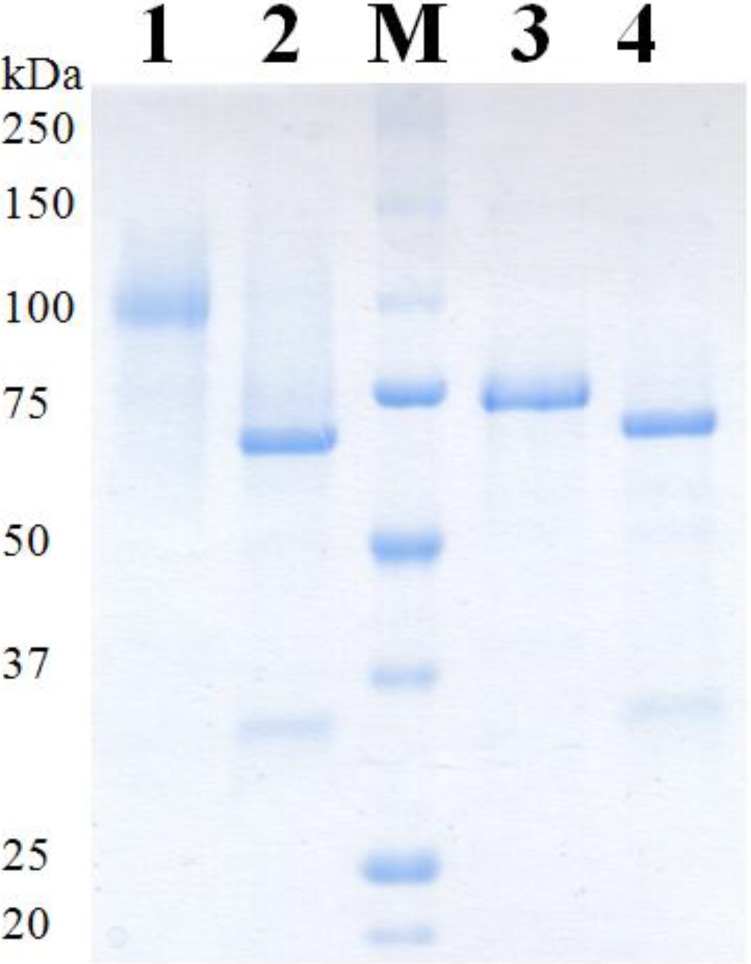
SDS-PAGE of purified PDHs. M, molecular marker; 1, AcPDH; 2, AcPDH deglycosylated; 3, Ax PDH; 4, AxPDH deglycosylated.

Recombinant AcPDH was purified from the cultivation broth in a four-step protocol (Table 1) including an additional hydrophobic interaction chromatography step (phenyl-source) compared to the three-step purification of AxPDH, which consisted of hydrophobic interaction chromatography, anion exchange chromatography and gel filtration as a polishing step. Before the AcPDH pool was loaded on the phenyl-source column, solid ammonium sulfate was added to a saturation of 40%, similar to the first purification step. Unexpectedly, the protein did not bind to the column in this case, and the whole PDH activity was found in the flow-through. This purification step indeed increased the specific activity from 0.4 U mg^−1^ to 2.8 U mg^−1^ but the flow-through still contained impurities due to the lack of restrictive pooling. Therefore, a subsequent gel filtration step was conducted, resulting in apparent homogeneity for both AcPDH and AxPDH (Figure 2), with final specific activities of 4.9 U mg^−1^ and 16.6 U mg^−1^, respectively. For AcPDH, two pools with different specific activities were formed due to rather low overall yields. Pool 1 represented the center of the elution peak and all further analyses were performed with this enzyme preparation. The low purification yield for AcPDH corresponds to the fact that PDH accounted only for 0.1% of the total protein in the extracellular fraction, whereas AxPDH accounted for more than 2%. The purification step that decreased the yield most dramatically is the first for both enzymes. As the starting volume for purification after centrifugation was around 4 L in both cases, the calculation of the total activity based on the activity per mL could lead to imprecise results. This is especially true in the case of AcPDH, where the volumetric activity per mL was below the detection limit of the standard ferrocenium/glucose activity assay and only represents an estimate. A reduction of the volume and therefore concentration of the cultivation broth would have been useful for more precise measurements of the initial volumetric activity. The higher degree of glycosylation of AcPDH could also play a disadvantageous role for the purification (Figure 2, Table 2). All concentrated protein pools showed the typical light yellow color of flavoproteins and were stable over several months at 4 °C in 65 mM sodium phosphate buffer pH 7.5. 

**Table 1:** Purification schemes of recombinant PDHs.

Purification step	Total protein [mg]	Total activity [U]	Specific activity [U mg^−1^]	Purification [-fold]	Yield [%]
**AcPDH**					
Crude extract	1730	154.4	0.1	1	100
Phenyl sepharose	124.4	27.4	0.2	2.5	18
DEAE sepharose	28.2	21.5	0.4	4.1	14
Phenyl source	5.5	15.2	2.8	31.0	10
Gel filtration pool 1	1.2	5.6	4.9	54.5	4
Gel filtration pool 2	0.4	1.5	3.7	41.9	1
**AxPDH**					
Crude extract	1226.4	1298.9	1.1	1	100
Phenyl sepharose	109.9	538.6	4.9	4.6	41
DEAE sepharose	40.2	524.9	13.1	12.3	40
Gel filtration	25.8	428.8	16.6	15.7	33

**Table 2:** Molecular properties of recombinant PDHs.

PDH	Mass SDS-PAGE [kDa]	Mass SDS-PAGE deglyc. [kDa]	Theor. mass [kDa]	Glycan mass [%]	*N*-Glyc sites predicted	*O*-Glyc sites predicted
Ac	98	68	61.8	31	6	3
Ax	73	68	62.3	7	5	0

### 2.5. Molecular Properties

**Figure 3:**
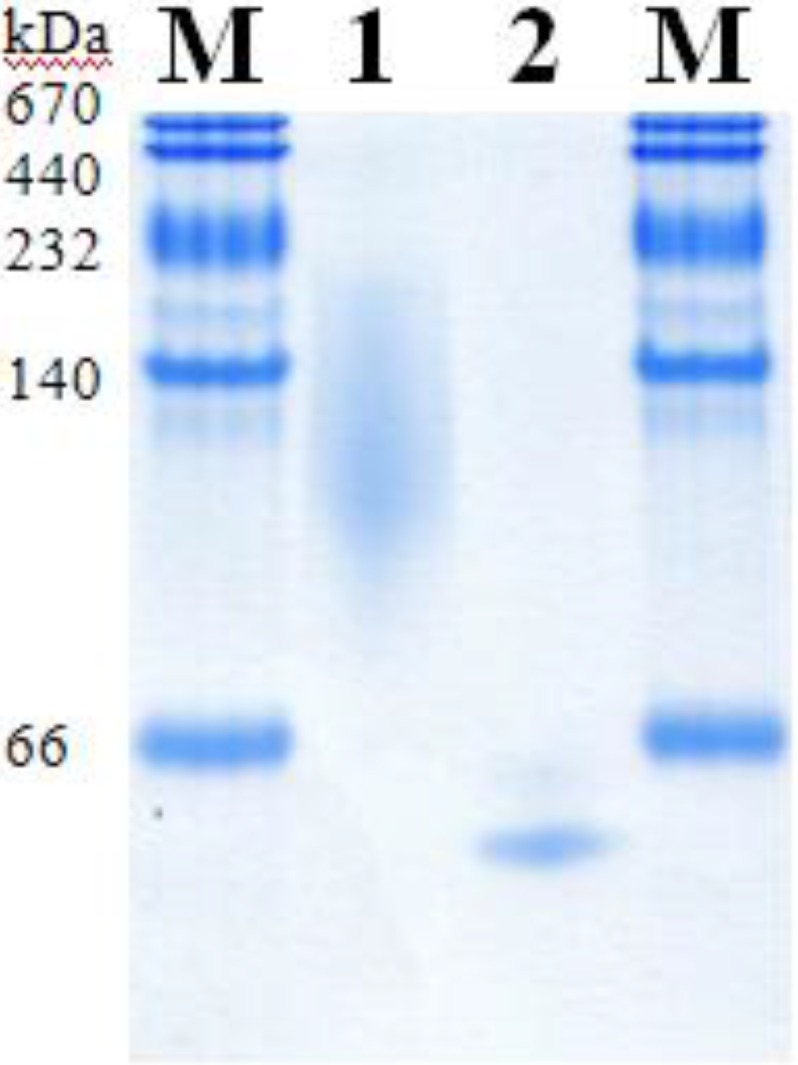
Native PAGE of purified PDHs. M, molecular marker; 1, AcPDH; 2, AxPDH.

The molecular masses of AcPDH and AxPDH were determined by SDS-PAGE (Figure 2) and native PAGE (Figure 3). AcPDH formed a diffuse band around 98 kDa (Figure 2, lane 1), after deglycosylation with PNGase F under denaturing conditions, a sharp band at 68 kDa could be observed (lane 2). AxPDH showed a band at 73 kDa (lane 4); after deglycosylation, the band shifted to around 68 kDa (lane 5). The high degree of glycosylation of AcPDH compared to AxPDH (Table 2) is also observed in native PAGE (Figure 3). From the migration difference of the glycosylated and deglycosylated protein band in the gel, a glycan mass of 31% for AcPDH and 7% for AxPDH could be calculated (Table 2). The native AxPDH [4] showed a slightly smaller mass on the SDS-PAGE compared to the recombinant protein (around 65 kDa). This is most likely due to a difference in glycosylation in basidiomycete fungi compared to the yeast *P. pastoris*. Whereas proteins in (homo) basidiomycete fungi carry *N*-glycans of the oligomannosidic type (4-9 mannoses), *P. pastoris* tends to produce hyper-mannosylated glycans [20,21,22]. The NetNGlyc 1.0 server found nine glycosylation motifs in the sequence of AxPDH, five of them putatively glycosylated, and nine for AcPDH with six of them likely to carry a glycan structure. Concerning *O*-glycosylation, NetOGlyc 3.1 server predicted three sites for AcPDH with potential above the threshold and none for AxPDH. The potentially higher degree of *O*-glycosylation of AcPDH compared to AxPDH cannot account for the large difference in glycan mass between the two proteins. The deglycosylation was carried out using PNGase F, which exclusively removes *N*-glycans, and the deglycosylated proteins have a quite similar molecular mass. Furthermore, *O*-glycans in *P. pastoris* mostly consist of up to three, rarely four, mannose units in contrast to hyper-mannosylated *N*-glycans [23]. A substantial over-glycosylation with a comparable glycan content of approximately 30% of protein expressed in *P. pastoris* was also observed for AmPDH [9]. PDH is one of the rare flavoproteins carrying a covalently bound cofactor [24]. Covalent incorporation was proven by the method of Scrutton [25], the flavin associated with the protein gives a fluorescent signal when exposed to UV-light. The positive control (AmPDH), AcPDH and AxPDH showed a bright band under UV-light whereas the negative control glucose oxidase (GOx) from *A. niger* [26] did not give any signal (Figure S2).

UV-Vis spectra of AcPDH and AxPDH in the oxidized state were recorded; typical flavoprotein absorbance maxima around 450 nm and 340 nm could be observed (Figure S3; AxPDH: data not shown).

### 2.6. Kinetic Properties

Catalytic constants for selected sugar substrates and electron acceptors were determined and are summarized in Table 3 and Table 4. For both enzymes, D-glucose represents the preferred electron donor. This is mainly due to the *K*_m_ value, which displays a more than 10 times higher affinity for this substrate compared to e.g., D-galactose. The pentose sugar D-xylose is the second best substrate for Ac and AxPDH. This finding stands in contrast to the catalytic efficiencies of other so-far characterized PDHs like from *Agaricus meleagris* [5] and the native *A. xanthoderma* PDH [4], where L-arabinose and D-galactose are preferred over D-xylose. The catalytic efficiency of AxPDH with D-xylose is more than 75% of the catalytic efficiency for the main substrate D-glucose, whereas for AcPDH it is only 13%. The *k*_cat_/*K*_m_-values of AcPDH are in general lower for all sugar substrates. Remarkable here is that the catalytic efficiencies of Ac and AxPDH are similar for lactose but the *K*_m_ of AcPDH is 5.5-times lower than the *K*_m_ of AxPDH, whereas the *k*_cat_ behaves the opposite way.

**Table 3:** Apparent kinetic constants for selected electron donors determined at 30 °C with 0.2 mM ferrocenium hexafluorophosphate as the electron acceptor.

	AcPDH			AxPDH		
	*K*_m_ [mM]	*k*_cat_ [s^−1^]	*k*_cat_/*K*_m_ [mM^−1^ s^−1^]	*K*_m_ [mM]	*k*_cat_ [s^−1^]	*k*_cat_/*K*_m_ [mM^−1^ s^−1^]
D-glucose	0.35 ± 0.06	4.10 ± 0.19	11.7	0.49 ± 0.03	13.02 ± 0.38	26.6
D-galactose	7.13 ± 0.19	5.23 ± 0.53	0.7	4.99 ± 0.16	24.77 ± 2.23	5.0
D-xylose	4.19 ± 0.26	6.37 ± 0.05	1.5	1.44 ± 0.07	29.16 ± 0.25	20.3
L-arabinose	4.23 ± 0.02	3.05 ± 0.04	0.7	4.16 ± 0.58	22.90 ± 2.23	5.5
Lactose	53.16 ± 0.10	3.12 ± 0.17	0.1	293.84 ± 8.00	24.65 ± 0.37	0.1

**Table 4:** Apparent kinetic constants for selected electron acceptors determined at 30 °C with 25 mM D-glucose as the electron donor.

	AcPDH			AxPDH		
	*K*_m_ [mM]	*k*_cat _ [s^−1^]	*k*_cat_/*K*_m _[mM^−1^ s^−1^]	*K*_m_ [mM]	*k*_cat_ [s^−1^]	*k*_cat_/*K*_m _[mM^−1^ s^−1^]
Fc^+^PF_6 _(pH 8.5)	1.19 ± 0.17	19.92 ± 2.87	16.7	0.03 ± 0.00	22.07 ± 0.05	735.7
1,4-BQ (pH 4)	0.12 ± 0.01	34.82 ± 1.02	302.8	3.25 ± 0.51	12.89 ± 1.56	4.0
DCIP (pH 4)	0.11 ± 0.00	10.56 ± 0.57	96.0	0.09 ± 0.01	7.65 ± 0.66	85.0

Concerning the kinetic constants for the electron acceptors, the two enzymes have quite different preferences (Table 4). For AcPDH 1,4-benzoquinone is the favored substrate whereas AxPDH shows a clear preference for ferrocenium hexafluorophosphate. All PDHs that have been characterized to date show a higher catalytic activity with ferrocenium compared to 1,4-benzoquinone; AcPDH is the first PDH where a clear preference for 1,4-benzoquinone was observed [4,5]. The catalytic efficiencies for DCIP are nearly equal for both PDHs. In general, PDH shows activity only with a limited group of electron acceptors whereas it oxidizes a very broad range of sugar substrates. Many major mono- and oligosaccharide components of lignocellulose can be utilized as substrates, giving evidence for the putative biological function of PDH in lignin degradation [7]. Recently, the molecular mechanism of glucose oxidation by *A. meleagris* PDH was explored using MD simulation; the findings support the experimentally observed promiscuity of PDH concerning sugars [27].

**Figure 4:**
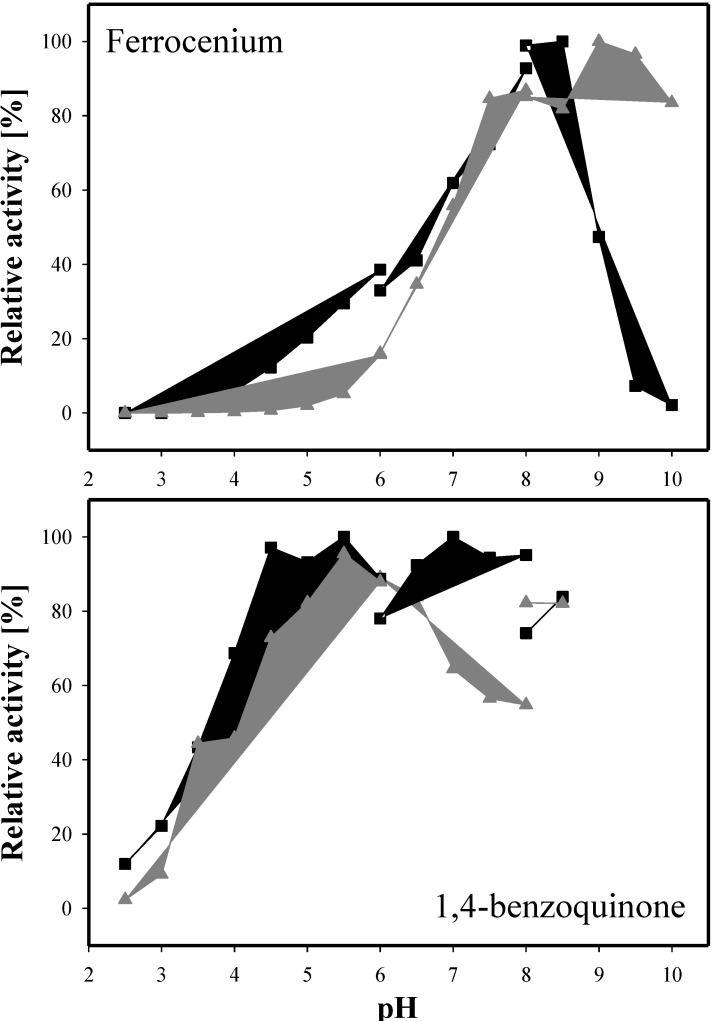
pH optima of AcPDH (black squares) and AxPDH (grey triangles) with the electron acceptors ferrocenium hexafluorophosphate and 1,4-benzoquinone; D-glucose as electron donor.

The pH dependence of PDH activity was tested for the electron acceptors ferrocenium hexafluorophosphate and 1,4-benzoquinone with D-glucose as the electron donor (Figure 4). Using 1,4-benzoquinone as the electron acceptor, AcPDH displayed maximum activity at pH 7 (phosphate buffer) and already reached 95% activity at pH 5.5 (citrate buffer). AxPDH showed the highest activity at pH 5.5 (citrate buffer). With ferrocenium hexafluorophosphate, the optimum pH for AcPDH was 8.5 and 9 for AxPDH (borate buffer). Compared to the native AxPDH [4], the pH optimum for ferrocenium hexafluorophosphate is comparable but with 1,4-benzoquinone the native AxPDH exhibited highest activity at pH 2.5 and around 70% activity at pH 8. The recombinant AxPDH showed less than 2.5% activity at pH 2.5. In general, it can be observed that the pH optimum is highly dependent on the electron acceptor used. PDHs from *Agaricus sp*. showed pH optima in the basic region when using ferrocenium hexafluorophosphate whereas with 1,4-benzoquinone the enzymes were more active under acidic conditions and in some cases showed a second maximum in the alkaline region, which could be due to high blank readings caused by the formation of quinhydrone [2,4,5].

### 2.7. Batch Carbohydrate Conversion Experiments

**Figure 5:**
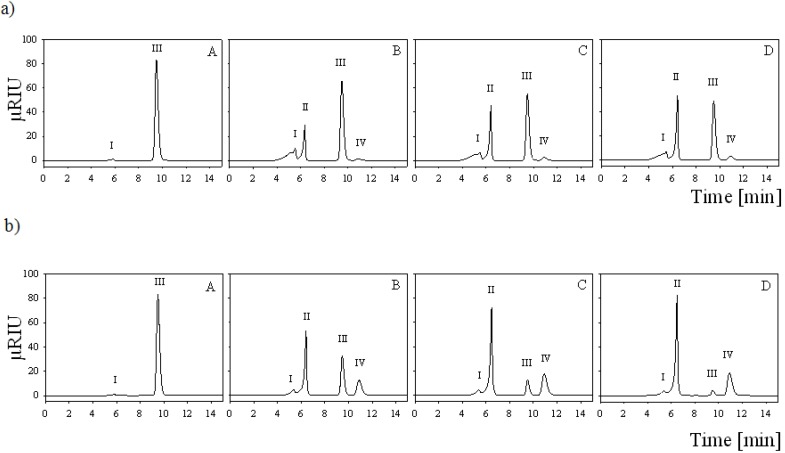
(**a**) HPLC analysis of lactose conversion by AcPDH; (**b**) and AxPDH at 0 h (A), 1 h (B), 3 h (C) and 7 h (D) incubation. Peaks: I, residual salt from enzyme preparation; II, lactobionic acid; III, lactose; IV, 2-dehydrolactose.

Both enzymes were used for the conversion of 25 mM lactose together with equimolar amounts of the electron acceptor 1,4-benzoquinone in 1 mL batch experiments and the reaction products were analyzed by HPLC (Figure 5). Conversions were carried out in water due to interference of buffer salts with the HPLC analysis. 10 U of AxPDH converted lactose to nearly 100% (97%), producing 69% (67%) lactobionic acid and 31% (30%) 2-dehydrolactose (Figure 5a). Due to the fact that the cultivation yield of AcPDH was quite low, only one unit of the purified protein was used for the conversion experiment. Therefore, only 48% of the lactose was converted and yielded 88% (42%) lactobionic acid and only 12% (6%) 2-dehydrolactose (Figure 5b). These results confirm the preference for C-1 oxidation in AcPDH-catalyzed conversions of lactose (the ratio of C-1 to C-2 oxidation is 7:1), compared to the AxPDH-catalyzed reaction where the ratio of C-1 to C-2 oxidation is only 2:1. C-2,3 oxidation, which can occur when oxidation at C-2 is complete under excess of 1,4-benzoquinone, could not be observed [12]. For industrial use, there is still need for improvement of the conversion yield. Acidification due to spontaneous hydrolysis of lactobionolactone to lactobionic acid slowed down the enzyme activity at the end of the conversion but could be avoided by buffering.

## 3. Experimental Section

### 3.1. Chemicals and Microorganisms

All chemicals, sugar standards for HPLC and media components were purchased from Sigma (Steinheim, Germany) unless otherwise stated and were of the highest purity available. Restriction endonucleases, T4 DNA ligase and Phusion High-Fidelity DNA Polymerase were obtained from Fermentas (St. Leon-Rot, Germany) unless otherwise stated and were used according to the manufacturer’s instructions. *Agaricus campestris* (strain CCBAS 20649) and *Agaricus xanthoderma* (strain CCBAS 225) were obtained from the Culture Collection of Basidiomycetes of the Academy of Sciences (Prague, Czech Republic). *Escherichia coli* strain NEB5α (New England Biolabs, Ipswich, MA, USA) was used for subcloning, *Pichia pastoris* strain X-33 (Invitrogen, Carlsbad, CA, USA) was used for expression. Ferrocenium hexafluorophosphate and the various substituted quinones were obtained from Aldrich (Steinheim, Germany). Phenyl-Sepharose Fast Flow resin was purchased from Amersham Pharmacia Biotech (Uppsala, Sweden), DEAE Sepharose Fast Flow resin and Sephacryl S300-HR resin were from GE Healthcare (Chalfont St. Giles, UK). GOx from *Aspergillus niger* was from Sigma.

### 3.2. Isolation of Genomic DNA and RNA

For DNA- and RNA-isolation, approximately 10 mL liquid Sabouraud medium was inoculated with mycelial fragments from freshly grown malt extract agar plates. The cultivations were performed in petri dishes at 25 °C without shaking over 3 weeks. Mycelia were harvested, squeezed dry between filter paper and shock-frozen in liquid nitrogen. Portions of approximately 100 mg of mycelium were used for DNA- and RNA-isolation. Genomic DNA extraction was performed according to Liu* et al.* [28]. Total RNA was extracted using Trizol reagent (Invitrogen) according to the manufacturer’s instructions. To remove genomic DNA, the samples were incubated with *DNAseI* as recommended by the manufacturer. The isolated mRNA was reverse-transcribed using RevertAid First Strand cDNA Synthesis kit (Thermo Fisher Scientific, Waltham, MA, USA) and the float primer (Table S1).

### 3.3. Cloning and Sequencing of AcPDH Encoding Gene

Nucleic acid amplifications were done using Phusion High-Fidelity Polymerase, GC-Buffer, dNTP mix, oligonucleotide primers (VBC Biotech, Vienna, Austria) and a BioRad C-1000 thermocycler (BioRad, Vienna, Austria). Nucleotide sequences of all primers used in this work are shown in Table S1. To obtain the PDH-encoding gene from *A. campestris* degenerate primers (AcPDHfwd1-3) were designed according to a sequence analysis of conserved regions in *pdh* genes from *A. meleagris* and *A. bisporus* and used for the amplification of cDNA fragments of various lengths. 35 PCR cycles at temperatures of 98 °C (10 s), 57 °C (20 s) and 72 °C (1 min) and an initial denaturation at 98 °C (2 min) and a final extension at 72 °C (7 min), were employed with cDNA as template and “universal” as reverse primer. The amplicons were purified and sequenced by a commercial sequencing service (LGC Genomics, Berlin, Germany). For identification of the 5‘ flanking region, including the native signal sequence, the DNA Walking SpeedUp Premix Kit (Seegene, Seoul, South Korea) was used. Target-specific reverse primers (AcTSP1-3) were designed, genomic DNA was used as template and the PCRs were done according to the manufacturer’s guidelines. The resulting PCR products were purified by using the illustra GFX PCR DNA and Gel Band Purification Kit (GE Healthcare) and sequenced. To obtain full-length cDNA clones a PCR was performed with the primer pair AcPDHfwd and AcPDHrev and cDNA as template. The resulting fragment was cloned into the pJET1.2 vector (Fermentas) according to the instructions in the manual and sequenced.

### 3.4. Cloning and Sequencing of AxPDH Encoding Gene

To obtain the 5‘- flanking region, including the native signal sequence, the DNA Walking SpeedUp Premix Kit (Seegene) was used. Target-specific reverse primers (AxTSP1-3) were designed according to *A. xanthoderma* PDH gene sequence, genomic DNA was used as template and the PCRs were done according to the manufacturer’s guidelines. The resulting PCR products were purified and sequenced. To obtain full-length cDNA clones a PCR was performed with the primer pair AxPDHfwd and AxPDHrev and cDNA as template. The amplified sequence was temporarily cloned into the pJET1.2 vector (Fermentas) according to the instructions in the manual and sequenced.

### 3.5. Construction of Expression Vectors for P. pastoris

The *acpdh* gene (in the pJET1.2 vector) was re-amplified with the primers AcPDHKpnIfwd and AcPDHNotIrev. The re-amplification of the *axpdh* gene was performed using the primer pair AxKpnIfwd and AxNotIrev. The resulting PCR products were digested with *KpnI* and *NotI* and ligated into the equally treated vector pPICZb (Invitrogen). After transformation into chemically competent *E. coli* NEB5α, according to the manufacturer’s instructions, the plasmids were proliferated, linearized with *SacI* and transformed into electrocompetent *P. pastoris*, which were prepared according to Lin-Cereghino* et al.* [29]. For selection the Luria Bertani (LB) medium contained 25 µg mL^−1^ Zeocin in case of *E. coli*, while the YPD-medium contained 100 µg mL^−1^. The resulting colonies were picked and grown in 96-well deep well plates.

### 3.6. Microscale Screening for High-Producing PDH Transformants

Microscale cultivation and expression in 96-well deep well plates was done according to Weis* et al.* [30] with some modifications. Cells were grown in 250 µL BMD1 (13.4 g·L^−1^ yeast nitrogen base, 0.4 mg·L^−1^ biotin, 10 g·L^−1^ D-glucose, 200 mM potassium phosphate [pH 6.0]) at 25 °C, 385 rpm and 60% humidity for approximately 60 h to reach the stationary growth phase. Induction was started by the addition of 250 μL of BMM2 medium (13.4 g·L^−1^ yeast nitrogen base, 0.4 mg·L^−1^ biotin, 1% methanol, 200 mM potassium phosphate [pH 6.0]) to reach a final concentration of 0.5% methanol. After 70, 82, and 108 h of incubation, 50 μL BMM10 (BMM2 with 5% methanol) were added to maintain inducing conditions. The cultivation was stopped after 130 h by centrifugation of the deep-well plates at 3000 rpm at room temperature for 20 min. PDH activity was measured using 2,6-dichloroindophenol (DCIP, ε520 = 6.8 mM^−1^ cm^−1^) as electron acceptor and D-glucose as donor. Fifty µL of the supernatant were transferred to the 96-well screening plates and the time-dependent reduction of 300 µM DCIP in 100 mM sodium acetate buffer pH 4 containing 50 mM D-glucose was followed at 520 nm with a PerkinElmer EnSpire plate reader. The reaction was started by addition of 150 µL of the DCIP assay mixture to the screening plate and end-point measurements were carried out after incubation at 30 °C for 2 and 4 h.

### 3.7. Sequence Analysis

The translated amino acid sequences of the obtained cDNAs were analyzed using the programs Translate, Compute pI/MW and SignalP at http://www.expasy.org/ [31]. A multiple sequence alignment of AmPDH1, AbPDH, AcPDH and AxPDH was created using the MUSCLE algorithm (EMBL-EBI, Cambridgeshire, UK). Sequence identities were determined by BLAST search [32]. Predictions for *N*- and *O*-glycosylation sites were performed on the NetNGlyc 1.0 Server and the NetOGlyc 3.1 Server [19] of the Center for Biological Sequence Analysis (CBS) at the Technical University of Denmark (http://www.cbs.dtu.dk/services/). All predicted *N*- and *O*-glycosylation sites with a threshold above 0.5 except for Asn-Pro-sites were displayed.

### 3.8. Nucleotide and Protein Sequence Accession Numbers

The NCBI accession numbers for the sequences in this work are: AY53306, AY753308, DQ117577, AAW82996, AAW82997, AAW82998, AAW82999, AAZ94874, AAZ94875 (*A. meleagris* PDHs); AY764148, AAW92124, EKV41672 (*A. bisporus* PDH); AY764147, AAW92123, KF534751 (*A. xanthoderma* PDH); KF534750 (*A. campestris* PDH).

### 3.9. Recombinant Protein Production in P. pastoris

AxPDH and AcPDH were produced in a 7-L bioreactor (MBR, Wetzikon, Switzerland) with an initial volume of 4 L basal salts cultivation medium, according to the “*Pichia* Fermentation Process Guidelines” (Invitrogen) with slight modifications. After autoclaving the bioreactor, the temperature was set to 30 °C, the pH was adjusted to 5 and maintained by addition of 28% ammonium hydroxide solution during the cultivation. Dissolved oxygen was regulated to 4% by supplying filtered air and adjusting the stirrer velocity (around 800 rpm). Two shaking flasks with 20 mL YPD-Zeocin medium each were inoculated with the colony-PCR-verified *P. pastoris* clones and grown overnight at 30 °C and 120 rpm. The cultures were transferred to two shaking flasks with 200 mL YPD medium each and again grown overnight at 30 °C and 120 rpm. This culture (400 mL) was used to inoculate the bioreactor. After consumption of the glycerol in the batch medium (indicated by an O_2_ spike), a feed of 50% glycerol containing 12 mL L^−1^ PTM1 trace salts was initiated with around 20 mL h^−1^ over night. Protein production was induced by changing to a feed of 100% methanol containing 12 mL PTM1 trace elements per liter. The feed rate was adjusted to maintain a dissolved oxygen concentration of around 4%. Samples were taken at least twice a day and biomass wet weight, PDH activity and total protein concentration was determined. The formation of foam was avoided by daily manual addition of approximately 10 mL a 10% antifoam 204 solution (Sigma). When no further increase in the specific activity could be observed, the bioreactor was harvested and the cultivation broth was centrifuged at 4 °C and 6000 rpm in a Sorvall Evolution RC centrifuge (Thermo Fisher Scientific).

### 3.10. Protein Purification

Solid ammonium sulfate was added to the cultivation supernatants to a saturation of 40%. The crude extracts (approximately 4 L) were applied to a 750 mL phenyl-sepharose FF column (GE Healthcare), and washed with binding buffer (50 mM potassium phosphate, pH 6.5 containing 1.5 M ammonium sulfate). The protein was eluted using a linear gradient from 0–100% elution buffer (50 mM potassium phosphate, pH 6.5) in 1 column volume (CV). Prior to the next purification step, the pooled fractions were desalted using cross-flow filtration (SpectrumLabs, Houston, TX, USA) to a conductivity equal to or less than 2 mS/cm. The pools of desalted fractions were loaded to a 60 mL DEAE sepharose column (GE Healthcare), washed with binding buffer (50 mM BisTris, pH 6) and eluted with a linear gradient from 0–100% elution buffer (50 mM BisTris, 1 M NaCl, pH 6) in 4 column volumes. For AcPDH, the concentrated pool from the anion exchange chromatography was subjected to a second hydrophobic interaction chromatography step using a 70 mL phenyl-source column (GE Healthcare). The purification was conducted similar to the first hydrophobic interaction chromatography step except for the elution of the protein, which was carried out in 5 CV. 

The fractions with the highest Ax- and AcPDH activities were pooled and concentrated to a volume of around 2 mL using an Amicon Ultra Centrifugal Filter Unit (EMD Millipore, Billerica, MA, USA). The concentrated pools were applied to a 190 mL Sephacryl S300 gel filtration column (GE healthcare) equilibrated with 50 mM potassium phosphate buffer (pH 7.5) containing 150 mM NaCl. Fractions with the highest purity were pooled, concentrated for buffer exchange (65 mM sodium phosphate buffer, pH 7.5) and stored at 4 °C.

### 3.11. Enzyme Assay, Molecular Properties

Standard PDH activity was measured by following spectrophotometrically the D-glucose dependent reduction of the ferrocenium ion (Fc+) to ferrocene at 300 nm and 30 °C as described before [4] with modifications: The standard reaction mixture (1 mL) contained 50 µmol sodium phosphate buffer pH 7.5, 0.2 µmol of ferrocenium hexafluorophosphate and 25 µmol D-glucose. Protein concentration was determined using the method of Bradford using a BSA standard curve and a prefabricated assay solution (BioRad). Enzymatic deglycosylation and SDS-PAGE were carried out as described in Sygmund* et al.*, using the Precision Plus Protein Unstained Standard (BioRad) [9]. 1.5–2 µg of the protein samples were loaded in each lane. Native PAGE was performed using 10% and 5% polyacrylamide as the separation and stacking gels, respectively, and Tris-glycine buffer (pH 8.3) as the electrode buffer [33]. 5–10 µg of the protein samples were loaded in each lane. Staining procedure was carried out using Bio-Safe Coomassie (BioRad) according to the manufacturer’s instructions. For determination of molecular weight, HMW Native Marker Kit (GE Healthcare) was used. To proof the covalent linkage of the FAD cofactor an additional SDS-PAGE was performed according to Scrutton [25]. 10 µg of the protein samples were loaded in each lane, the covalently linked FAD was visualized by exposure of the gel to UV-light (λ 302 nm, GelDoc2000, BioRad). As a positive control, recombinant *A. meleagris* PDH was loaded [1]; glucose oxidase from *A. niger* was used as a negative control.

Molecular masses of the proteins were calculated from their migration distances on the SDS-PAGE; the theoretical mass was derived from ExPASy ProtParam tool (http://web.expasy.org/protparam/) [31]. The glycan mass in% was calculated from the difference of the masses of the glycosylated and deglycosylated proteins on the SDS-PAGE.

UV-Vis absorbance spectra of 13 µM AcPDH and AxPDH were recorded in 65 mM sodium phosphate buffer pH 7.5 at room temperature using a U-3000 spectrophotometer (Hitachi, Tokyo, Japan) from 300–700 nm.

### 3.12. Kinetic Properties

Apparent kinetic constants for electron donors were measured using the standard activity assay with ferrocenium hexafluorophosphate as described above. Kinetic constants for ferrocenium hexafluorophosphate, 1,4-benzoquinone and 2,6-dichloroindophenol were determined using 25 mM D-glucose as electron donor. The observed data were fitted to the Michaelis-Menten equation and kinetic constants were calculated by nonlinear least-squares regression. Using the molecular mass, turnover numbers (*k*_cat_) and catalytic efficiencies (*k*_cat_/*K*_m_) were calculated. The pH optima with the electron acceptors ferrocenium hexafluorophosphate (0.2 mM) and 1,4-benzoquinone (2 mM) were determined with the following buffers: 100 mM citrate (pH 2.5–6), 100 mM potassium phosphate (pH 6–8) and 100 mM borate (pH 8–10) and 25 mM D-glucose as the electron donor. Activities with 1,4-benzoquinone were not determined above pH 8.5 due to the formation of quinhydrone under basic conditions.

### 3.13. Batch Conversion Experiments

Small-scale lactose conversions were carried out in 1.5 mL Eppendorf vials containing 25 mM 1,4-benzoquinone, 25 mM lactose monohydrate and 1 U of purified AcPDH or 10 U of purified AxPDH in 1 mL deionized water. The vials were incubated at 30 °C and 400 rpm in a thermomixer, samples were taken in regular time intervals (50 µL). Immediately after sampling, PDH activity was stopped by heating the sample to 99 °C for 3 min. The samples were centrifuged, diluted 1:2 and subjected to HPLC analysis.

### 3.14. HPLC Analysis of Batch Conversion Products

HPLC analysis of the batch conversion products was performed on a Dionex Summit HPLC system (Thermo Fisher Scientific) fitted with a Shodex RI-101 refractive index detector (Shoko Scientific, Yokohama, Japan) using an Aminex HPX 87-K column (BioRad) with a guard column. Samples and standards were eluted at 80 °C with deionized water (0.5 mL min^−1^). For the calculation of lactose and lactobionic acid concentrations, standards were included in the run. As there was no standard available for 2-dehydrolactose, the ratio of 2-dehydrolactose to lactobionic acid was estimated by comparing the peak areas.

## 4. Conclusions

This study demonstrates the successful expression of the PDH-encoding genes from the litter-degrading basidiomycetes *A. campestris* and *A. xanthoderma*, in the eukaryotic host organism *P. pastoris*. Small-scale conversion experiments with lactose as substrate revealed that AcPDH has a strong preference for C-1 oxidation, resulting in the production of lactobionic acid. Compared to AxPDH, which produces mixtures of C-2/C-1 oxidation products in a 1:2 ratio, AcPDH is a very attractive biocatalyst for the production of lactobionic acid. Further research towards a better expression yield is required for industrial applications/purposes.
